# Cellular Structures for Electric Vehicle Battery Systems: A Critical Review and Design Guidelines

**DOI:** 10.3390/ma19142985

**Published:** 2026-07-10

**Authors:** Alessandra Ceci, Girolamo Costanza, Maria Elisa Tata

**Affiliations:** Department of Industrial Engineering, University of Rome “Tor Vergata”, Via del Politecnico 1, 00133 Rome, Italy; alessandra.ceci@uniroma2.it (A.C.); elisa.tata@uniroma2.it (M.E.T.)

**Keywords:** cellular structures, lattice, honeycomb, foam, TPMS, auxetic, energy absorption, crashworthiness, electric vehicle, battery pack protection

## Abstract

Architected cellular materials are increasingly proposed for electric-vehicle (EV) battery systems as lightweight solutions that can combine crashworthiness, intrusion mitigation and, in some cases, thermal functionality. However, the literature remains fragmented across heterogeneous architectures, metrics and test conditions, which often prevents design-oriented comparison and limits transferability to pack-level implementation. This review consolidates the state of the art on cellular structures for EV battery applications, covering foams, honeycombs, lattice/TPMS architectures and auxetic or bio-inspired concepts. A unified, design-oriented comparative framework is introduced to discuss mechanical performance (e.g., specific energy absorption, peak crushing force, deformation control), thermal considerations and manufacturing–scalability constraints under an EV battery-pack perspective. By explicitly linking cellular architecture selection to pack safety and integration requirements, including limited crush space, joining interfaces, enclosure stiffness and emerging “pack-as-a-structure” concepts, this work translates dispersed results into actionable engineering insights. Finally, decision-oriented guidelines and a selection flowchart are proposed to support early-stage architecture choice and highlight the most critical research gaps, particularly in multiphysics validation, standardized benchmarking and production-ready integration.

## 1. Introduction

The rapid development of electric vehicles (EVs) is driven by the need to reduce greenhouse gas emissions and improve energy efficiency in the transportation sector [1]. Within this framework, the battery pack represents the dominant subsystem in terms of both mass and design constraints, directly affecting vehicle range, safety, chassis architecture and structural integration. Current technological trends and open challenges in EV development have been extensively discussed in literature, encompassing battery safety concerns [2,3,4,5], thermal management strategies [6,7,8], and mechanical integrity under abuse scenarios [9,10,11].

Battery safety has become a critical concern, particularly due to thermal runaway and thermal runaway propagation (TRP), which may be triggered by thermal overloads, electrical faults, or mechanical abuse such as impacts, intrusion and local deformation [2,3,4,5,6]. Mechanical loading can damage separators and current collectors, potentially leading to internal short circuits and rapid temperature rise [3]. These issues highlight the need for integrated multi-physics design approaches accounting simultaneously for mechanical, thermal and electrochemical phenomena.

Battery Thermal Management Systems (BTMS) play a key role in maintaining temperature uniformity and mitigating the onset or propagation of thermal runaway. Several solutions, including liquid cooling, phase change materials (PCM) and hybrid strategies, have recently been investigated [7,8].

From a mechanical perspective, experimental abuse tests such as nail penetration, blunt rod compression and impact loading have been widely employed to characterize cell failure mechanisms [9,10,11,12,13]. These studies clearly demonstrate that mechanical protection of the battery pack is essential for overall vehicle safety.

Such requirements are further enforced by regulatory and certification frameworks, including United Nations Economic Commission for Europe (UNECE) Regulation R100, the International Organization for Standardization (ISO) Standard 6469-1:2019, and Underwriters Laboratories (UL) Standard 2580:2020, which define system-level safety and abuse testing procedures with specific crush force, thermal, electrical, and mechanical shock testing requirements.

In this context, cellular structures emerge as promising multifunctional solutions due to their high specific energy absorption, tunable mechanical behavior through architectural design and potential to combine structural and thermal functions. This review therefore aims to critically assess and compare such architectures, identifying their advantages, limitations, and suitability for EV battery system applications.

### Scope and Methodology

This work is conceived as a critical, narrative review rather than a systematic review, and does not follow a formal PRISMA protocol. The literature was retrieved from Scopus, Science Direct and Google Scholar, using combinations of the keywords like cellular structures, lattice, honeycomb, foam, TPMS, auxetic, energy absorption, crashworthiness, electric vehicle, battery pack protection. The search covered publications from 1997 to 2026, with emphasis on contributions published in the last eight years to reflect the current state of the art, while a limited number of seminal works (e.g., the foundational Gibson–Ashby formulation) were included regardless of date for their theoretical relevance. Studies were included when they addressed the mechanical, thermal or manufacturing behavior of cellular architectures relevant to automotive or EV battery applications, and were excluded when they dealt with cellular materials in unrelated fields or lacked quantitative performance indicators suitable for the comparative framework adopted here. Priority was given to peer-reviewed journal articles, complemented by a restricted set of standards and conference papers where directly relevant. The selected works were then organized according to architecture type and analyzed under a unified set of performance metrics (Section 2), which forms the basis for the comparative analysis and design guidelines presented in the following sections.

Figures 2 and 3 were prepared with the assistance of Google Gemini 3 Pro, using its image-generation functionality indicated by the interface as “Nano Banana Pro”. The tool was used only to support the graphical rendering and visual organization of illustrative figures. It was not used to generate scientific data, experimental results, quantitative analyses, literature selection, interpretation of results, or conclusions. The final figures were manually reviewed by the authors to verify the accuracy of the scientific content, terminology, spelling, and grammar. The authors also verified that the figures do not include copyrighted third-party material, logos, or other elements un-suitable for publication in an open-access journal.

## 2. Theoretical Foundations of Cellular Structures

Cellular structures, also referred to as cellular or architected materials, are materials characterized by an intentionally designed micro- or meso-scale porous architecture designed to achieve tailored mechanical and physical properties. Unlike bulk solids, the performance of cellular materials does not only depend on the base material but is strongly governed by cell geometry, relative density and overall structural architecture [14,15].

Cellular materials include open- and closed-cell foams, honeycombs, periodic lattice structures, and more complex architected configurations obtained through advanced design approaches. In these systems, cell topology and geometry represent additional design variables, enabling the modulation of stiffness, strength, energy absorption capability and thermal conductivity [14,16,17].

The mechanical behavior of cellular structures is classically described by the Gibson–Ashby model, which establishes scaling relationships between the properties of a cellular material and those of its parent solid as a function of relative density [18,19]. The relative density is defined as in Equation (1):

(1) $$\bar{\rho }=\frac{\rho^{*}}{\rho_{s}}$$

where $\rho^{*}$ is the density of the cellular structure and $\rho_{s}$ is the density of the solid material. According to the Gibson–Ashby framework, the elastic modulus $E^{*}$ and the collapse (yield) stress $\sigma^{*}$ scale with relative density through power-law relationships of the form given in Equations (2) and (3).

(2) $$\frac{E^{*}}{E_{s}}=C_{1}{\left(\frac{\rho^{*}}{\rho_{s}}\right)}^{n}$$

(3) $$\frac{\sigma_{pl}^{*}}{\sigma_{ys}}=C_{2}{\left(\frac{\rho^{*}}{\rho_{s}}\right)}^{m}$$

where $C_{1}$ and $C_{2}$ are dimensionless constants of order unity and the exponents n and m reflect the dominant deformation mechanism of the cell walls or struts. For bending-dominated architectures, such as open-cell stochastic foams, the cell walls deform predominantly in flexure, yielding n=2 and m=3/2. For stretching-dominated architectures, such as the octet-truss lattice, the struts are loaded axially and the scaling becomes linear, with n=m=1. This distinction provides the theoretical basis for the higher mass efficiency of stretching-dominated lattices: at equal relative density, their stiffness decays only linearly rather than quadratically with $\bar{\rho }$, which directly motivates the architecture-selection trends discussed in Section 3 [18,20].

Under quasi-static or dynamic compressive loading, many cellular structures exhibit a characteristic stress–strain behavior consisting of three stages: (i) an initial elastic regime, (ii) a collapse plateau associated with progressive cell buckling or yielding and (iii) a densification stage where collapsed cells come into contact and stiffness increases rapidly [15,17]. The plateau region is of particular relevance for crashworthiness applications, as it allows large amounts of energy to be absorbed at approximately constant stress levels.

To quantitatively assess the energy absorption performance of cellular structures, normalized crashworthiness indicators are commonly employed. The Specific Energy Absorption (SEA) is defined as the absorbed energy per unit mass and, for a specimen compressed up to the densification strain $\varepsilon_{d}$, is computed as in Equation (4)

(4) $$SEA=\frac{1}{\rho^{*}}\int_{0}^{\varepsilon_{d}}\sigma (\varepsilon )\;d\varepsilon$$

In parallel, the Peak Crushing Force (PCF) corresponds to the maximum force recorded during deformation and is a critical safety indicator, since high peak forces may induce damage to protected components or passengers [17,21,22].

Beyond mechanical properties, the thermal behavior of cellular structures is strongly influenced by architectural features. The effective thermal conductivity, $k_{\mathrm{eff}}$, depends on relative density, cell connectivity and the continuity of the solid phase. In general, open-cell metallic foams and lattice structures exhibit higher values of $k_{\mathrm{eff}}$ compared to closed-cell foams or honeycomb structures, making them attractive for multifunctional applications combining energy absorption and thermal management [8,9,15].

Based on the theoretical framework discussed above, the different cellular architectures reviewed in this work are compared using a consistent set of mechanical, geometric and thermal performance indicators. These metrics, summarized in Table 1, include relative density, SEA, PCF, plateau stress, densification strain and effective thermal conductivity. This unified set of indicators enables a quantitative and objective comparison across different cellular concepts and forms the basis for the comparative analysis and design guidelines presented in the following sections [15,17,21,22].

## 3. Cellular Structures: Types, Properties and General Design

**Figure 1:**
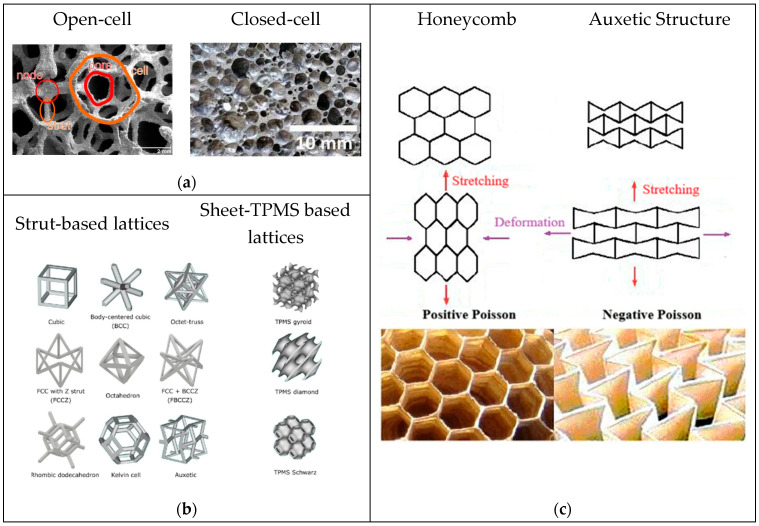
(**a**) Stochastic foam structures, characterized by open- and closed-cell random morphology [26,27]; (**b**) Strut-based lattices and Sheet-TPMS based lattices; (**c**) honeycomb structures with periodic prismatic cells and anisotropic mechanical response and auxetic structures exhibiting negative Poisson’s ratio behavior and enhanced load redistribution.

Cellular structures encompass a broad class of materials characterized by a porous or cellular architecture specifically designed to achieve lightweight behavior combined with tailored mechanical and physical properties. Although these categories are widely used in the literature, their performance in EV battery systems is not determined by topology alone. Relative density, deformation mode (stretching- vs. bending-dominated), manufacturing route and integration constraints (space claim, joining, thermal pathways, and serviceability) often govern real-world effectiveness more than the nominal architecture. Therefore, the following classification is used not as a rigid taxonomy, but as an engineering lens to discuss performance trends and practical trade-offs. Based on geometry, topology and deformation mechanisms, cellular structures employed in engineering applications can be broadly classified into foams, honeycombs, lattice structures and more general architected materials [14,16,23]. This classification provides a useful framework to understand their mechanical behavior, manufacturability and suitability for automotive and electric vehicle (EV) applications. The main classes of cellular architectures considered in this review are schematically illustrated in Figure 1.

Foams consist of a stochastic network of cells and are commonly divided into open-cell and closed-cell configurations. Their isotropic nature and relatively simple manufacturing routes make foams attractive for applications requiring uniform mechanical response and effective thermal management. However, their random microstructure often leads to limited tunability and scatter in mechanical behavior, making performance tuning less controllable than in periodic architectures, a critical limitation when design margins are tight at battery-pack level [14,15].

Honeycombs are characterized by a periodic array of prismatic cells, typically with hexagonal geometry. They exhibit high in-plane stiffness and excellent specific energy absorption, but their mechanical response is strongly anisotropic. As a result, honeycombs are widely used in energy absorbers and sandwich cores, particularly in automotive and aerospace structures, but require careful orientation with respect to loading direction [14,17]. In EV battery protection, this anisotropy is both an advantage and a limitation: honeycombs can be extremely efficient when the impact direction is well-defined, but their performance may degrade under complex multiaxial loading and non-ideal boundary conditions typical of pack integration.

Lattice structures are periodic, truss- or surface-based architectures composed of interconnected struts or continuous surfaces. Unlike foams and honeycombs, lattice structures offer a high degree of geometric flexibility, enabling systematic optimization of stiffness, strength and energy absorption through unit-cell design. Recent advances in additive manufacturing have significantly expanded their applicability in lightweight and crash-relevant components [16,23,24,25]. Nevertheless, the strong dependence on unit-cell geometry and manufacturing quality implies that reported high performance is not automatically transferable to production-ready components without addressing defect sensitivity, surface finish and repeatability.

Architected cellular materials have demonstrated superior fatigue resistance and energy absorption efficiency compared to conventional cellular systems, at the expense of increased design and manufacturing complexity [15,23]. Within this class, auxetic concepts, despite their promising load redistribution capability, often face a manufacturability–complexity constraint and their benefits should be evaluated against simpler architectures under the same normalization and boundary conditions [28,29].

The mechanical behavior of cellular structures is governed by relative density, cell geometry, and deformation mode. Depending on architecture, collapse may occur through elastic buckling, plastic yielding, or progressive fracture of cell walls and struts. These mechanisms directly influence the stress–strain response, the stability of the collapse plateau and the overall energy absorption capability [15,17].

Crashworthiness performance is typically evaluated in terms of specific energy absorption (SEA), peak crushing force (PCF) and collapse stability. Honeycombs and lattice structures generally provide higher SEA compared to stochastic foams, while architected lattices allow further enhancement through controlled collapse modes and graded designs. However, increased performance often comes with trade-offs in terms of manufacturability and cost, which are particularly relevant for large-scale automotive deployment [17,23].

For automotive and EV applications, fatigue performance is a critical but often overlooked aspect. Architected cellular materials have shown promising fatigue resistance due to distributed stress paths and the ability to delay crack initiation through geometry-driven mechanisms. Nevertheless, fatigue life remains strongly dependent on manufacturing quality, surface defects and relative density, especially for additively manufactured lattice structures [15].

From a design perspective, the choice of cellular architecture represents a multi-criteria problem involving trade-offs between energy absorption efficiency, structural anisotropy, durability, and manufacturing feasibility. In the context of EV battery systems, no cellular architecture can be considered intrinsically optimal. The apparent advantage of a given topology in terms of specific energy absorption can be significantly reduced when pack-level constraints are introduced, including enclosure stiffness, limited crush space, joining interfaces and the coexistence of structural and thermal functions. Architecture selection should therefore be requirement-driven: directional crash absorbers may favor honeycomb-like concepts, multifunctional structural enclosures may benefit from lattice or TPMS-based approaches and localized cell protection may justify auxetic or bio-inspired solutions, provided that scalability and variability are explicitly accounted for.

## 4. Cellular Structures for Automotive and EV Applications

Cellular structures have long been employed in the automotive sector to achieve lightweight components with high energy absorption capability. Typical applications include crash boxes, energy absorbers, structural reinforcements and sandwich panel cores, where mass reduction must be balanced against stringent passive safety requirements. Numerous studies have shown that the geometry and relative density of cellular structures strongly influence collapse mechanisms and crashworthiness performance [17,30,31].

While cellular structures have been extensively and successfully employed in conventional automotive applications, their direct transfer to electric vehicle platforms is not straightforward. The introduction of the battery pack as a dominant mass, volume and safety-critical component fundamentally alters crash scenarios, load paths and packaging constraints. As a result, cellular structures in EVs are required not only to absorb energy, but also to control localized deformation and protect electrochemically sensitive components.

In electric vehicles, the battery pack imposes additional and often conflicting requirements on structural design, including: (i) limited available crush space, (ii) stringent mass constraints to preserve driving range, (iii) the need to limit peak loads and local deformation near battery cells and (iv) compatibility with thermal management systems and enclosure architectures. These constraints significantly reduce the design freedom typically available in conventional automotive energy absorbers.

In conventional vehicles, cellular structures are primarily used as dedicated energy absorbers designed to dissipate impact energy away from critical components. Honeycomb structures, in particular, have been widely adopted due to their high specific energy absorption and relatively stable collapse response. Bio-inspired optimization of honeycomb geometries has been shown to further enhance energy absorption while reducing peak crushing forces [21]. However, unlike conventional crash components designed to dissipate energy away from occupants, EV battery protection requires strict limitation of intrusion and deformation in proximity to the battery cells, where even moderate local strains may trigger safety-critical failure mechanisms.

From a critical perspective, this transition highlights that cellular structures in EVs cannot be designed as generic energy absorbers. Instead, their role shifts toward controlled deformation, intrusion mitigation and system-level compatibility with the battery pack. Architectures that are effective in conventional automotive applications may therefore become suboptimal or even unsuitable when applied to EV battery systems without adaptation. This shift motivates the need for EV-specific investigations, which are discussed in the following section with a focus on battery pack protection.

**Figure 2:**
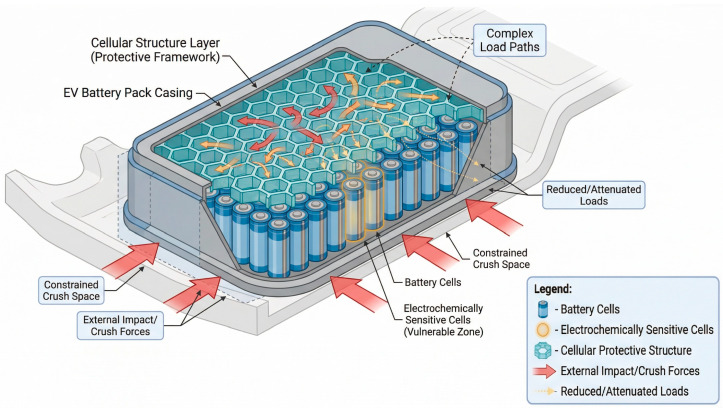
Conceptual system-level representation of battery pack protection requirements in electric vehicles. Cellular structures integrated within the battery pack act as protective layers that redistribute and attenuate external mechanical loads (represented by vertical arrows indicating impact direction), limiting local intrusion and deformation of electrochemically sensitive cells under constrained crush space and complex load paths. The protective cellular layer is schematically shown in cross-section, illustrating load distribution to the enclosure structure.

To clarify the specific protection challenges associated with electric vehicle battery systems, a system-level conceptual representation is introduced in Figure 2. The figure summarizes the key constraints governing battery pack protection, including limited crush space, complex load paths and the need to control local intrusion and deformation in proximity to electrochemically sensitive cells. The vertical arrows in Figure 2 represent external mechanical impact forces acting on the battery pack. The protective cellular structure, integrated as a load-bearing layer, redistributes and attenuates these external forces, thereby minimizing stress transmission to the electrochemically sensitive battery cells. This load-redistribution function is essential to prevent internal short circuits and mechanical damage that could trigger thermal runaway, while simultaneously managing the constrained crush space typical of EV pack-level integration.

The cellular protective layer illustrated in Figure 2 must simultaneously distribute external impact loads and limit intrusion to electrochemically sensitive cells. Based on the performance ranges in Table 3, honeycomb structures (SEA 15–35 J g^−1^, PCF ratio 1.3–1.8) or low-density foams (SEA 3–15 J g^−1^) are typical choices for the primary protective layer, with material thickness typically ranging from 10 to 30 mm depending on crush space constraints. The selection prioritizes stable plateau stress behavior (σ_peak_/σ_plateau_ close to 1) to ensure consistent load attenuation and minimize peak force transmission to battery cells. Aluminum alloys (Al3003-H16, Al3104-H19) or titanium-based foams are preferred for their thermal conductivity and crashworthiness performance, though cost-performance trade-offs often favor conventional aluminum honeycombs for production-scale implementation.

## 5. Cellular Structures for Battery Pack Protection in EVs

The protection of electric vehicle battery packs represents one of the most critical challenges in EV structural design. Unlike conventional automotive components, battery packs contain electrochemically sensitive elements whose mechanical damage can result in severe safety consequences, including internal short circuits and thermal runaway. As a result, battery pack protection requires not only effective energy absorption, but also strict control of local deformation, intrusion and peak loads transmitted to the cells.

In recent years, a growing body of research has investigated the use of cellular structures to enhance the mechanical protection of EV battery packs. These studies explore a wide range of architectures, including honeycombs, lattice-based materials, bio-inspired designs and auxetic structures, typically focusing on impact, intrusion, or quasi-static crushing scenarios. Most contributions rely on numerical simulations to demonstrate improved energy absorption, reduced peak forces and enhanced deformation control compared to conventional solutions.

It should be noted that the emphasis in this section on honeycomb-derived, bio-inspired, and auxetic structures reflects the current state of the literature, where these architectures have received substantially more experimental and numerical investigation for EV battery pack protection applications. While conventional lattice topologies and TPMS structures demonstrate significant potential, their application to battery protection remains less extensively explored in peer-reviewed literature, with most studies focusing on aerospace and general crashworthiness contexts rather than EV-specific requirements. This coverage imbalance mirrors the current research emphasis and highlights an important gap for future investigations.

**Figure 3:**
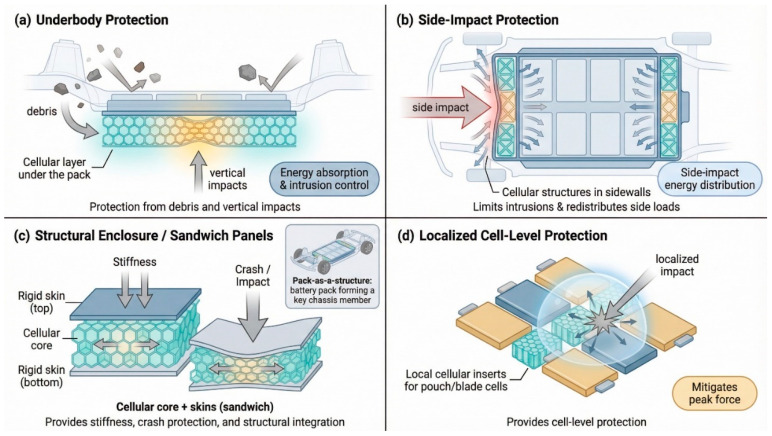
Schematic overview of typical integration strategies of cellular structures within electric vehicle battery systems: (**a**) underbody protection layers for vertical impact and debris intrusion mitigation; (**b**) side-wall integration to limit lateral intrusion and redistribute impact loads; (**c**) structural battery enclosures and sandwich panels combining cellular cores with load-bearing skins; (**d**) localized cellular inserts providing targeted protection of electrochemically sensitive cells. Different integration locations correspond to distinct functional roles and design requirements.

Cellular structures can be integrated into electric vehicle battery systems at different locations, each associated with distinct functional roles and protection strategies. To clarify these integration approaches, Figure 3 provides a schematic overview highlighting underbody protection, side-impact mitigation, structural enclosure solutions, and localized cell-level protection.

This predominance of idealized numerical modeling has been noted in recent critical assessments of battery protection strategies [23]. Most investigations focus on component-level performance without explicitly modeling cell-to-cell interaction, enclosure stiffness coupling, or post-impact thermal evolution [22,23]. As a consequence, reported performance gains at the component level may not directly translate into improved battery safety under realistic EV pack-level operating conditions.

Figure 3 shows four representative integration strategies, each with distinct mechanical and manufacturability requirements:

(a) Underbody protection: Honeycomb or foam layers (10–20 mm thickness, SEA 15–25 J g^−1^) positioned beneath the battery pack to absorb vertical impacts and debris intrusion. This configuration prioritizes cost-effectiveness and scalability, typically using extruded aluminum honeycomb.
(b) Side-impact protection: Cellular structures integrated into sidewalls (15–40 mm thickness) to redistribute lateral loads. Lattice structures (SEA 20–60 J g^−1^) or TPMS-based architectures offer superior performance here but at higher manufacturing cost; simpler honeycomb solutions remain viable for moderate impact scenarios.
(c) Structural battery enclosure (sandwich panels): Cellular cores bonded between rigid skins (total thickness 30–50 mm). This multifunctional approach combines stiffness, crash protection and thermal pathways. TPMS and lattice structures excel in this application despite higher cost, as they enable simultaneous optimization of mechanical and thermal performance.
(d) Localized cell-level protection: Thin cellular inserts (5–15 mm) positioned directly around pouch or blade cells. Auxetic structures (SEA 5–40 J g^−1^, σ_peak_/σ_plateau_ 0.8–1.2) are particularly effective here due to their stable load distribution and reduced peak force transmission, mitigating internal short circuits under localized crushing. Material selection depends on production volume, thermal requirements and regulatory targets, with trade-offs between performance, cost and manufacturability guiding the final choice.

Bio-inspired cellular architectures have attracted increasing attention for EV battery pack protection due to their ability to combine high energy absorption efficiency with controlled collapse mechanisms and reduced structural mass. A recent comprehensive review systematically investigated the use of bio-inspired cellular structures for EV battery pack protection, highlighting how nature-derived architectures can significantly enhance crashworthiness by controlling deformation propagation and limiting loads transmitted to battery cells [22].

Several numerical and experimental studies further support these findings. In an extensive parametric investigation, 45 different nature-inspired cellular configurations were analyzed, demonstrating that appropriate topology selection can lead to specific energy absorption values as high as approximately 35 J g^−1^, together with a significant reduction in loads transmitted to the battery enclosure [32]. Similarly, the application of bio-inspired honeycomb structures to a battery pack system resulted in up to 30% reduction in global deformation and approximately 10% decrease in peak stress compared to conventional configurations, highlighting the potential of such architectures in mitigating mechanical damage to battery cells [33].

It is important to note, however, that the reported improvements for bio-inspired structures should be interpreted with appropriate context. Most of the results cited above, particularly the 35 J g^−1^ values and the 30% deformation reduction, derive predominantly from numerical finite element simulations rather than full-scale experimental validation [32,33]. Additionally, the 30% deformation reduction may be strongly dependent on the specific direction of impact loading, with performance potentially varying considerably when the cellular structure is subjected to impacts from different orientations or oblique angles. As with other numerically optimized architectures, these findings require validation through comprehensive experimental testing at the battery pack system level, particularly under multi-directional and realistic loading scenarios.

Beyond bio-inspired concepts, auxetic cellular structures characterized by a negative Poisson’s ratio have been proposed as particularly effective solutions for pouch cell protection, due to their ability to laterally expand under compression and redistribute loads more uniformly. Recent studies combining machine learning techniques with multi-objective optimization (ML + NSGA-II) enabled the design of optimized auxetic structures, achieving absolute SEA values of approximately 40 J g^−1^, about 13 times higher than the carbon-steel Re-entrant baseline (3 J g^−1^) [34]. It should be noted, however, that this improvement results from the combined optimization of material (from carbon steel to Al6061-T6), unit-cell shape (Re-entrant to Star-shaped) and wall thickness (from 1 to 2.95 mm), rather than from a same-material, same-mass comparison; the transition to aluminum alone accounts for a density reduction of approximately 2.9 times, which inherently boosts mass-normalized metrics such as SEA.

The performance of cellular structures for battery pack protection has also been investigated through explicit dynamic crash simulations. Studies integrating honeycomb and lattice-based cellular structures within battery pack models demonstrated effective reduction in peak loads and improved deformation distribution under representative impact scenarios [35]. Nevertheless, these investigations remain predominantly numerical and require further experimental validation at the system level.

The main quantitative outcomes of representative studies on cellular structures for EV battery pack protection are summarized in Table 2.

From a critical perspective, the reviewed studies clearly demonstrate that cellular structures can significantly enhance the mechanical protection of EV battery packs, particularly by reducing localized deformation and peak loads transmitted to battery cells. Bio-inspired and auxetic architectures show remarkable energy absorption capability and effective deformation redistribution under impact and intrusion scenarios.

However, most of the reported improvements are obtained under idealized numerical conditions, often neglecting realistic pack-level constraints such as enclosure stiffness, cell-to-cell interaction, joining interfaces and thermal management components. Moreover, performance metrics are frequently reported without consistent normalization, making direct comparison among different architectures difficult.

From an engineering standpoint, these limitations suggest that the adoption of advanced cellular architectures should be driven by local protection requirements rather than by global performance optimization. Highly optimized bio-inspired or auxetic structures are particularly suitable for intrusion-prone regions, whereas simpler and more scalable solutions may be preferable for global pack protection.

## 6. System Integration: Cell Architecture, Battery Pack and EV Chassis

The integration of cellular structures within electric vehicle battery systems represents a critical design challenge that extends beyond the optimization of individual components. Unlike conventional automotive energy absorbers, battery packs are highly integrated systems in which structural, thermal, electrical and manufacturing requirements are tightly coupled. As a result, the effectiveness of cellular structures must be evaluated at the system level, considering their interaction with the battery enclosure, cell arrangement, thermal management system and vehicle architecture [23].

One of the key integration aspects concerns the role of the battery pack within the vehicle structure. The increasing adoption of pack-as-a-structure and cell-to-pack concepts implies that the battery enclosure contributes directly to global vehicle stiffness and load transfer. Real-world examples include Tesla’s structural battery packs in Model Y and Cybertruck platforms, and BYD’s cell-to-pack Blade Battery architecture integrated in production vehicles (BYD Han EV, Qin Plus EV, Toyota bZ3) [36,37]. In this context, cellular structures integrated into the battery pack must simultaneously provide crash protection, structural support and compatibility with load-bearing functions, rather than acting as standalone energy absorbers [38,39,40].

Cell format and packing strategy strongly influence the integration of cellular architectures. Pouch cells, prismatic cells and cylindrical cells exhibit different sensitivities to localized deformation and peak loads, which in turn affect the suitability of specific cellular solutions. For instance, auxetic and bio-inspired architectures may be particularly advantageous for pouch cell protection due to their ability to redistribute loads and limit local strains, whereas more conventional honeycomb or lattice-based solutions may be preferable for global structural reinforcement [22,41].

Thermal management represents an additional and often conflicting constraint. Cellular structures introduced for mechanical protection may alter heat dissipation paths, airflow and coolant routing within the battery pack. Materials and architectures optimized for energy absorption are not necessarily optimal from a thermal standpoint and excessive insulation or obstruction of thermal pathways may compromise battery performance and safety [3].

Manufacturability and assembly constraints further affect system-level integration. While advanced lattice-based and bio-inspired architectures offer high performance potential, their implementation at battery-pack scale raises challenges related to production rate, cost, quality control and joining with conventional metallic or composite enclosures. Tolerances, surface finish and repeatability become critical factors, particularly when cellular structures are intended to interact directly with battery cells or cooling components [23].

From a system integration perspective, these considerations highlight that cellular structures should not be optimized in isolation. Instead, their design must be embedded within a holistic framework that accounts for mechanical performance, thermal behavior, manufacturing feasibility and vehicle-level constraints. This system-oriented viewpoint is essential to translate the promising results reported at component level into viable and safe solutions for electric vehicle battery systems.

## 7. Advanced Design and Optimization Strategies

The high geometric flexibility of cellular structures makes them inherently suitable for advanced design and optimization strategies. Unlike conventional materials, the mechanical performance of cellular architectures can be significantly enhanced by tailoring topology, relative density distributions and hierarchical features. This design freedom is particularly valuable for electric vehicle (EV) applications, where multiple and often conflicting requirements, such as high energy absorption, peak load mitigation and mass reduction, must be satisfied simultaneously.

A wide range of optimization methods has been applied to cellular structures, including topology optimization, graded design strategies and hierarchical architectures. Pan et al. provided a comprehensive review on the design and optimization of uniform and non-uniform lattice structures, demonstrating that spatial variation of geometric parameters can substantially improve mechanical performance compared to homogeneous designs [16,42,43]. These approaches enable the redistribution of material to promote controlled collapse mechanisms and enhanced energy absorption efficiency.

Similarly, Nazir et al. presented an extensive overview of optimization strategies for cellular structures, with particular emphasis on manufacturability considerations associated with additive manufacturing. Their review highlights the importance of coupling computational design methods with manufacturing constraints in order to enable the practical realization of optimized cellular architectures at component and system level [14].

In automotive and EV applications, crashworthiness optimization is typically formulated as a multi-objective problem aimed at maximizing specific energy absorption (SEA) while minimizing peak crushing force (PCF). Within this framework, several advanced optimization concepts have been proposed to balance competing performance objectives. A notable example is the Hybrid Optimized Topological and Hierarchical (HOTT) cellular structure, which combines topological refinement with hierarchical design features and has demonstrated effective multi-objective optimization, leading to improved SEA and enhanced collapse stability [44,45].

Comparable optimization approaches have been applied to auxetic cellular structures for battery protection by combining machine learning techniques with NSGA-II-based multi-objective optimization. These studies demonstrated that data-driven optimization can effectively identify high-performance auxetic configurations, leading to exceptionally large improvements in specific energy absorption compared to baseline designs. The results highlight the potential of auxetic architectures when geometric parameters and deformation mechanisms are systematically optimized rather than selected through heuristic design [34].

Another representative approach is provided by bio-inspired Hybrid Topological Cellular Honeycomb Structures (HTCHS), which have been optimized using multi-objective particle swarm optimization (MOPSO). In these studies, the combined use of bio-inspired geometry and evolutionary optimization enabled significant enhancements in energy absorption performance compared to conventional honeycomb designs. The results further confirm that bio-inspired cellular architectures can greatly benefit from systematic multi-objective optimization frameworks when applied to crash-relevant EV components [46].

The increasing complexity of optimization problems has promoted the adoption of machine learning-based surrogate models to reduce computational cost. Artificial neural networks (ANNs) have been successfully employed to predict the energy absorption performance of bio-inspired lattice structures fabricated by additive manufacturing, enabling efficient exploration of large design spaces without resorting to exhaustive finite element simulations [47].

Surrogate models facilitate the integration of geometric optimization with additional design constraints and can significantly accelerate the exploration of high-dimensional design spaces. However, their reliability strongly depends on the quality and representativeness of the training data, as well as on the availability of experimental validation. Sensitivity to manufacturing defects, material variability and assembly tolerances remains a critical challenge, particularly for data-driven optimization strategies intended for industrial-scale EV applications.

Overall, advanced design and optimization strategies are essential to fully exploit the potential of cellular structures in EV applications. The integration of multi-objective optimization, advanced optimization algorithms and machine learning enables the development of high-performance architectures that are difficult to achieve using traditional design approaches. Nevertheless, the increasing model complexity highlights the need for common benchmarking criteria, consistent performance normalization and systematic experimental validation. These aspects are explicitly addressed in the subsequent comparative analysis presented in this review.

## 8. Manufacturability and Scalability

The adoption of cellular structures in electric vehicle applications is not governed solely by mechanical performance, but is strongly constrained by manufacturability, reliability and industrial scalability. While advanced cellular architectures can deliver exceptional energy absorption and deformation control at component level, their practical implementation must account for production feasibility, quality consistency and cost-effectiveness at automotive scale.

Additive manufacturing (AM) enables the fabrication of highly complex cellular and lattice architectures that are difficult or impossible to realize using conventional manufacturing routes. This capability has significantly expanded the design space for optimized and graded cellular structures. However, AM also introduces challenges related to defect control, surface quality, fatigue performance, repeatability and production cost. Metal AM processes such as Selective Laser Melting and Electron Beam Melting offer unprecedented geometric freedom, yet structural integrity and fatigue behavior are strongly influenced by process-induced defects and surface roughness, as highlighted by Benedetti et al. [15,48].

While AM excels in producing highly optimized, architected lattices for high-performance and low-volume applications, traditional manufacturing routes remain more suitable for large-scale automotive deployment. In particular, honeycomb-based solutions manufactured through extrusion, forming, or bonding processes offer a favorable balance between mechanical performance, cost and scalability, making them attractive for industrial implementation in EV platforms [16,49,50].

Scalability and producibility therefore remain major challenges for highly complex cellular geometries. Nazir et al. emphasized that manufacturability constraints should be considered from the earliest design stages, especially when cellular structures are intended for structural or safety-critical functions [13]. From an EV-specific perspective, Komara et al. further highlighted that manufacturability considerations play a decisive role in determining which cellular solutions can realistically be integrated into battery systems, where reliability, robustness, and cost constraints are particularly stringent [23].

Overall, additive manufacturing provides significant opportunities for the development of high-performance cellular structures and rapid design iteration. Nevertheless, the industrial adoption of cellular architectures in electric vehicles requires a careful balance between achievable performance gains and manufacturability, reliability and scalability constraints. Addressing these aspects is essential to ensure that optimized cellular designs can successfully scale from laboratory-scale demonstrations to robust, production-ready solutions for EV battery systems.

## 9. Comparative Analysis of Cellular Architectures

The literature reviewed in the previous sections clearly demonstrates the variety and diversity of cellular architectures proposed for automotive and electric vehicle (EV) applications. However, meaningful comparison among these solutions is often hindered by heterogeneous performance metrics, inconsistent loading conditions and different levels of experimental validation. As a result, reported performance advantages are frequently architecture- and study-dependent, limiting their direct applicability to engineering design decisions.

**Figure 4:**
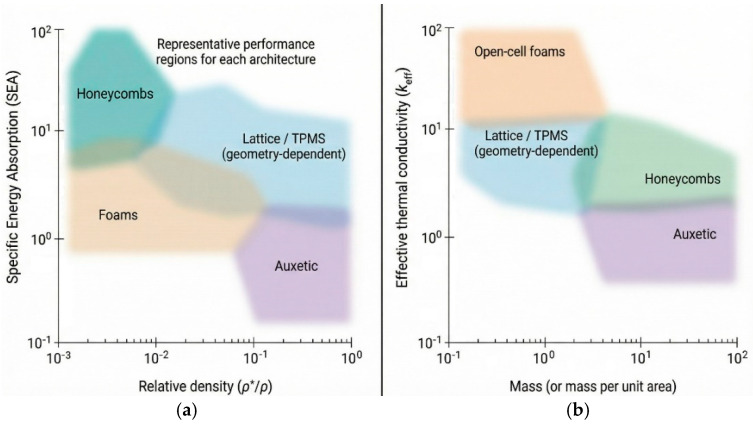
Ashby-type performance maps illustrating representative performance regions of the main cellular architectures considered in this review: (**a**) specific energy absorption as a function of relative density; (**b**) effective thermal conductivity as a function of mass. Colored regions indicate qualitative performance trends derived from the reviewed literature and are intended to support design-oriented comparison rather than quantitative benchmarking.

To support a design-oriented comparison and visualize the inherent trade-offs among different cellular architectures, Ashby-type performance maps are introduced in Figure 4. These graphical frameworks enable visual identification of performance regions and highlight the multidimensional nature of architecture selection, where no single structure is universally optimal. In the context of EV battery applications, two maps are particularly relevant:

(i) specific energy absorption versus relative density, illustrating the mass efficiency of different architectures;
(ii) effective thermal conductivity versus mass, showing the potential for multifunctional thermal-mechanical integration.

Honeycombs typically occupy regions of high mass efficiency and stable energy absorption; foams dominate thermally efficient regions; while lattice and auxetic architectures populate high-performance zones characterized by superior energy absorption but greater variability due to geometric and manufacturing sensitivity.

Figure 4 presents Ashby-type performance maps as qualitative schematic illustrations of performance trends and architectural trade-offs, not as quantitative benchmarking charts. The maps are intended to support conceptual design-oriented comparison; specific quantitative values, numerical ranges, and corresponding units [J g^−1^, W m^−1^ K^−1^, ρ*/ρs] are provided in Table 3 and should be referenced for precise engineering data.

Within these performance regions, the Ashby-type maps provide a visual framework for understanding trade-offs between energy absorption and material efficiency. To address these limitations, this section provides a comparative analysis of the main classes of cellular architectures, honeycombs, foams, lattice-based structures and auxetic architectures, using a unified and design-oriented set of indicators. The comparison explicitly considers mechanical performance (specific energy absorption, peak crushing force), thermal functionality, mass efficiency, manufacturability and cost, with particular focus on EV battery pack requirements.

A comparative overview of representative performance ranges for the main cellular architectures is reported in Table 3, with indicative numerical intervals compiled from experimental and numerical studies in the reviewed literature. The selected performance indicators include relative density range (ρ*/ρs), specific energy absorption (SEA) at 50% strain, peak-to-plateau stress ratio (σ_peak_/σ_plateau_) as a dimensionless proxy for collapse stability, effective thermal conductivity (k_eff_), relative manufacturing cost, and manufacturability. Reported intervals should be interpreted as the envelope of typical values documented in the literature for each architecture, rather than absolute upper and lower bounds, given the intrinsic scatter of cellular materials and the heterogeneity of test protocols. The peak-to-plateau stress ratio threshold of 1.5 is presented as an engineering guideline to distinguish between stable (bend-dominated) and unstable (stretch-dominated) collapse modes rather than as an absolute quantitative criterion, and reflects practical considerations for EV battery pack protection under multi-impact scenarios.

The SEA values reported in Table 3 are compiled from heterogeneous literature sources and may use different normalization methods. Most values are normalized to apparent (bounding-box) mass, but some original studies may report SEA normalized to solid material mass. This difference is critical: for structures with low relative density (ρ*/ρs < 0.1), normalizing to solid material mass instead of apparent mass can increase SEA values by 2–5×. Direct quantitative comparison between values from different sources should be done with extreme caution. Values should be interpreted as representative ranges from literature rather than absolute or directly comparable metrics. For rigorous energy absorption comparisons, researchers should refer to the original studies and verify the specific normalization method used in each case.

From a mechanical perspective, honeycomb structures consistently exhibit specific energy absorption in the range 15–35 J g^−1^ under out-of-plane quasi-static compression, with moderate peak-to-plateau stress ratios (1.3–1.8) that reflect their stable and predictable collapse behavior. It should be noted that the upper range of effective thermal conductivity for aluminum honeycombs reported in Table 3 may be conservative. Experimental studies on Al3003-H16 and Al3104-H19 honeycomb configurations have reported higher effective thermal conductivity values, particularly in the axial direction, reaching approximately 4–5 W/(m·K) [54]. This variation reflects differences in cell orientation, alloy composition, and measurement conditions, and reinforces the importance of directional characterization for thermally critical EV applications. Their established industrial use, manufacturing cost approximately 3–5× lower than metallic lattices produced via additive manufacturing, and extensive supply chain maturity make them a reference solution for crashworthiness applications.

Foams, particularly open-cell configurations, provide a more isotropic mechanical response and superior effective thermal conductivity, making them attractive when thermal management and heat dissipation play a dominant role. Nevertheless, their moderate energy absorption efficiency compared to periodic architectures restricts their use in applications where maximum crash energy dissipation is required.

Lattice-based and TPMS-inspired architectures offer the highest degree of performance tunability among the considered solutions. By tailoring unit-cell geometry and relative density, these architectures can achieve very high specific energy absorption while simultaneously enabling multifunctional integration, such as structural support combined with thermal or packaging functions. Despite these advantages, their widespread adoption remains constrained by high manufacturing costs, process variability and limited industrial maturity for large-scale EV production.

Auxetic architectures emerge as particularly promising for localized battery protection, especially for pouch and blade cell configurations. Their negative Poisson’s ratio behavior promotes load redistribution and reduces stress concentration, resulting in excellent control of localized deformation and peak loads. However, the geometric complexity of auxetic designs and the lack of scalable manufacturing routes currently limit their application to targeted protection zones rather than global pack-level solutions.

The ranges reported in Table 3 are consistent with recent experimental measurements performed by the present authors on metallic cellular architectures produced through different manufacturing routes. Quasi-static compression tests on aluminum honeycomb panels and closed-cell aluminum foams, reported by Ceci et al. [51], yielded specific energy absorption values at 50% strain of approximately 28 J g^−1^ for honeycombs and 20–25 J g^−1^ for closed-cell foams, consistently falling within the ranges of 15–35 J g^−1^ and 5–25 J g^−1^ reported in Table 3, respectively. For TPMS-based architectures produced via the Lost-PLA investment casting route in AA6082 aluminum alloy [52], a specific energy absorption of approximately 26 J cm^−3^ was measured, corresponding to ≈25 J g^−1^ when normalized by the apparent density (ρ* ≈ 1.04 g cm^−3^). This value falls in the lower portion of the 20–80 J g^−1^ range reported for TPMS lattices in Table 3, reflecting the contribution of casting-induced porosity (defect-related porosity ≈ 7%) to the local load-bearing cross-section of the struts, compared with defect-free AM counterparts. These direct measurements confirm that the ranges adopted in the comparative framework are representative of the experimental envelope and further highlight the sensitivity of SEA to manufacturing route, even for nominally equivalent architectures.

To visualize the inherent trade-offs between performance and lightweight efficiency, Ashby-type material selection maps provide a useful graphical framework. In the context of EV battery applications, two maps are particularly relevant: specific energy absorption versus relative density and effective thermal conductivity versus mass. Within these maps, honeycombs typically lie in regions of high mass efficiency and stable energy absorption, foams dominate thermally efficient regions, while lattice and auxetic architectures populate high-performance zones characterized by superior energy absorption but greater variability due to geometric and manufacturing sensitivity. These maps should be interpreted in terms of performance regions rather than discrete points, capturing the intrinsic scatter of architected materials.

Beyond mechanical protection, thermal management represents a critical secondary function of cellular structures in EV battery packs. Battery thermal runaway is a self-sustaining chain reaction initiated when internal temperatures exceed critical thresholds that vary by chemistry. For conventional NMC (Ni-Mn-Co) chemistry, exothermic reactions begin around 100 °C with separator melting occurring at 130–140 °C [3,6], while thermal runaway can propagate catastrophically at 150–180 °C and beyond [3]. Once initiated, thermal runaway propagates from cell to cell within milliseconds [6], making temperature uniformity and heat dissipation essential to safety. The effective thermal conductivity (k_eff_) of cellular structures, reported in Table 3, directly influences their ability to redistribute localized heat and maintain temperature uniformity across the pack. Open-cell metallic foams (k_eff_ = 5–15 W/(m·K)) and TPMS-based lattices (k_eff_ = 5–25 W/(m·K)) exhibit substantially higher thermal conductivity compared to closed-cell foams (k_eff_ = 2–8 W/(m·K)) or honeycombs (k_eff_ = 0.5–5 W/(m·K)), making them particularly attractive for thermally critical applications [7,8]. However, mechanical protection and thermal management often involve competing design objectives. Structures optimized for maximum energy absorption may obstruct heat dissipation pathways or restrict coolant circulation, potentially creating thermal gradients and hot spots adjacent to battery cells [7]. Consequently, multifunctional design strategies must explicitly balance crashworthiness with thermal performance, rather than treating these functions independently. Sandwich panel configurations with open-cell foam or TPMS cores bonded between rigid aluminum skins can simultaneously provide structural stiffness, mechanical impact protection and enhanced thermal conductivity along preferential heat transfer paths [7,8]. These hybrid approaches are emerging as promising solutions that address the multiphysics requirements of modern EV battery systems.

Overall, the comparative analysis confirms that no single cellular architecture can be considered universally optimal for EV applications. Instead, architecture selection is governed by fundamental trade-offs between crashworthiness and thermal functionality, performance, cost, and industrial scalability. Architectures delivering the highest mechanical performance are often those associated with the greatest manufacturing complexity and cost, whereas industrially mature solutions offer robustness and scalability at the expense of ultimate performance.

From an engineering standpoint, these results emphasize that cellular architecture selection should be driven by system-level requirements rather than by the maximization of a single performance metric. Honeycombs provide robust and cost-effective solutions for global energy absorption, foams are advantageous when thermal management is critical, while lattice-based and auxetic architectures are best suited for multifunctional or localized protection strategies. These insights form the foundation for the design-oriented guidelines and selection framework presented in the following section.

## 10. Engineering Design Guidelines

The comparative analysis presented in the previous sections demonstrates that the selection of cellular architectures for electric vehicle (EV) battery systems cannot be driven by a single performance metric. Effective design decisions must instead account for multiple and often competing requirements, including mechanical protection, thermal functionality, mass efficiency, cost constraints and industrial feasibility. This section translates the insights derived from the reviewed literature and the comparative assessment into practical, design-oriented engineering guidelines to support architecture selection in EV battery systems.

From a requirement-driven perspective, different cellular architectures emerge as optimal solutions depending on the dominant functional objective. When maximum energy absorption and stable crash response are required, conventional honeycomb structures and auxetic architectures represent the most suitable options. Honeycombs provide well-established and scalable solutions with predictable collapse behavior, while auxetic architectures offer superior load redistribution and peak force mitigation, particularly advantageous for pouch and blade cell protection.

In applications where thermal dissipation and thermal runaway mitigation are critical, open-cell metallic foams provide a favorable solution due to their high effective thermal conductivity and isotropic behavior. Their ability to combine mechanical compliance with enhanced heat transfer makes them particularly attractive for thermally critical regions of the battery pack.

For components requiring a high stiffness-to-weight ratio or multifunctional structural integration, lattice-based and TPMS-inspired architectures offer significant advantages. These architectures enable precise tuning of stiffness, strength and energy absorption at minimal mass, making them suitable for structural battery enclosures and load-bearing elements. However, their application should be carefully balanced against manufacturing complexity and cost.

In cost-sensitive scenarios and large-volume EV production, conventional honeycomb structures remain the most viable solution. Their low manufacturing cost, high repeatability and established industrial supply chains make them particularly suitable for global energy absorption and enclosure reinforcement.

Multifunctional requirements, involving simultaneous mechanical protection, stiffness contribution, and thermal functionality, can be effectively addressed through sandwich configurations combining structural skins with lattice- or foam-based cores. Such solutions provide a balanced compromise between performance, integration capability, and manufacturability.

These considerations highlight that cellular architecture selection should always be requirement-driven rather than performance-driven in isolation. To support early-stage design decisions, a system-level selection framework is proposed. The decision-making process should follow five key steps:

(i) Identification of the dominant functional requirement (e.g., crash protection, thermal management, stiffness, cost or multifunctionality);
(ii) Definition of secondary constraints, including mass budget, allowable peak crushing force, thermal limits and expected production volume;
(iii) Assessment of manufacturability constraints, such as available manufacturing routes, scalability, tolerances and cost;
(iv) Selection of candidate cellular architectures based on comparative performance trends;
(v) Refinement of the selected architecture through advanced optimization strategies, including graded density design, topology optimization and machine learning-assisted approaches.

**Figure 5:**
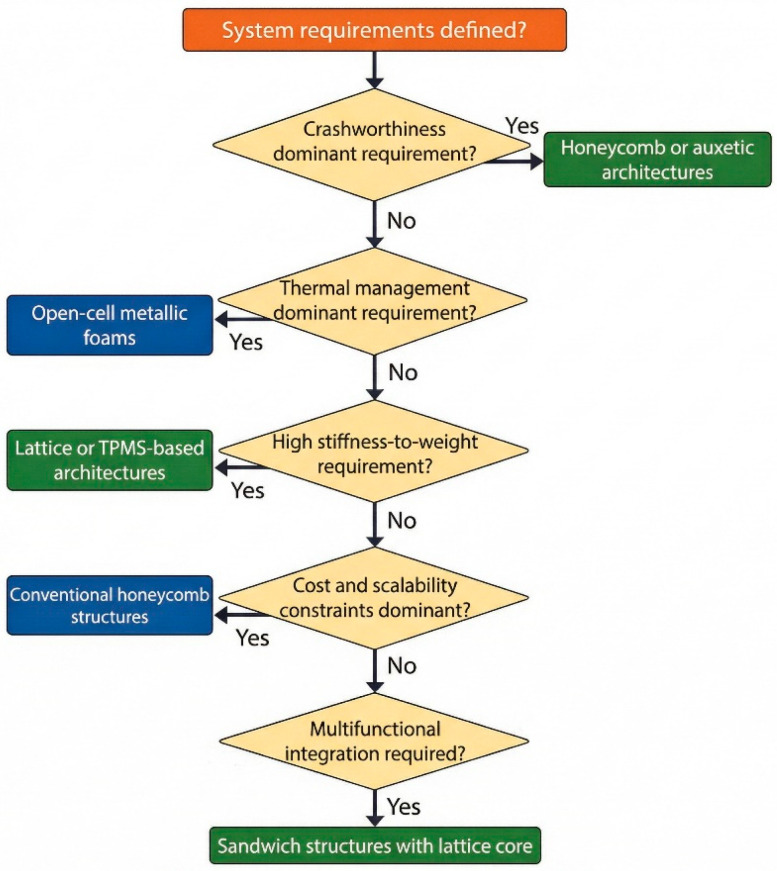
Design-oriented decision flowchart for selecting cellular architectures in electric vehicle battery systems. The flowchart guides architecture selection based on dominant functional requirements, including crashworthiness, thermal management, stiffness-to-weight ratio, cost and scalability constraints, and multifunctional integration.

This structured workflow ensures consistency between system-level requirements and architectural choices and can be directly represented through a design-oriented selection flowchart (Figure 5).

From an engineering standpoint, several practical recommendations emerge. Cellular structures should be designed as integral components of the battery system rather than as add-on crash absorbers. Performance optimization must explicitly consider peak crushing force and deformation control in addition to specific energy absorption. Highly optimized and complex architectures should be reserved for critical intrusion-prone regions, such as side-impact zones or underbody protection areas, while simpler and more scalable solutions are preferable for global pack-level protection. Manufacturing constraints should be evaluated from the earliest design stages to avoid non-scalable or economically unfeasible solutions. Finally, multifunctional designs should explicitly address mechanical–thermal coupling rather than treating structural and thermal functions independently.

While Figure 5 provides a structured starting point for architecture selection, it is important to recognize that this sequential decision framework represents a simplified view of the design process. In practice, EV battery pack protection rarely involves a single dominant objective; instead, engineers must simultaneously satisfy multiple competing requirements: crashworthiness, thermal management, weight efficiency, cost constraints, and manufacturability. The proposed guidelines should therefore be interpreted as an initial screening framework rather than prescriptive rules. Real-world architecture selection requires explicit trade-off analysis among competing objectives. For example, honeycomb structures may offer superior cost-effectiveness and scalability but may sacrifice thermal management capability compared to open-cell foams or TPMS-based solutions. Similarly, lattice or auxetic architectures may deliver exceptional energy absorption and load control but at significantly higher manufacturing cost and complexity. The application of these guidelines depends on design-specific assumptions regarding loading scenarios, manufacturing routes, vehicle architecture, regulatory requirements and cost targets, and must therefore be adapted to specific EV platforms and project constraints. Designers should use Figure 5 in conjunction with the comparative performance data in Table 3 to identify candidate architectures, then employ weighted scoring or multi-objective optimization methods to navigate the trade-off space based on project-specific priorities.

Overall, this section translates the findings of the literature review and comparative analysis into actionable engineering design guidelines. By explicitly linking cellular architecture selection to functional requirements, manufacturability and system-level constraints, the proposed framework provides a practical pathway toward the effective and realistic integration of cellular structures in electric vehicle battery systems.

## 11. Open Challenges and Future Perspectives

Despite the significant progress achieved in the design and application of cellular structures for electric vehicle (EV) battery systems, several open challenges and research gaps remain that currently limit their widespread industrial adoption. This section critically discusses the main limitations of the state of the art and outlines key directions for future research.

A major limitation of the existing literature is the strong predominance of numerical studies based on finite element modeling. Most investigations are conducted on simplified geometries and idealized boundary conditions, focusing on component-level performance. While such studies provide valuable insight into collapse mechanisms and energy absorption behavior, experimental validation remains limited and full-scale battery pack or vehicle-level testing is rare. This imbalance raises concerns regarding the transferability of many proposed solutions to real-world EV applications [17,22,23,33,34].

Another important gap is the lack of standardized design criteria and structural guidelines specifically addressing battery pack protection strategies based on cellular architectures. Unlike conventional automotive crash components, cellular-based battery protection solutions are often developed on a case-by-case basis, without unified performance metrics, safety margins, or accepted testing protocols. This fragmentation hinders objective comparison among architectures and slows industrial adoption.

The limited integration of multiphysics modeling approaches represents a further critical challenge. Most existing studies primarily address mechanical crashworthiness, while the coupled interaction between mechanical deformation, thermal behavior and electrochemical response remains insufficiently explored. However, battery safety is inherently governed by the interaction among these domains, particularly in scenarios involving cell deformation, internal short circuits and thermal runaway initiation and propagation [2,5,22,38,55,56,57]. Neglecting these couplings may lead to overly optimistic assessments of protection effectiveness.

Future research should therefore move toward fully coupled mechanical–thermal–electrochemical modeling frameworks capable of capturing both short-duration crash events and longer-term post-impact thermal and electrochemical evolution. Such approaches are essential to realistically assess battery pack safety and to design truly multifunctional protective structures.

A further cross-cutting limitation of the reviewed literature concerns the predominance of quasi-static mechanical characterization. Most reported stress–strain responses and specific energy absorption values for cellular architectures are obtained at strain rates in the range $\;\dot{\varepsilon }$ ≈ 10^−3^–10^−1^ s^−1^, whereas automotive crash events relevant to EV battery systems typically involve effective strain rates of $\;\dot{\varepsilon }$ ≈ 10^1^–10^2^ s^−1^, with localized intrusion scenarios that may locally reach $\;\dot{\varepsilon }$ ≈ 10^3^ s^−1^. In cellular materials this gap is non-trivial, as the dynamic response deviates from the quasi-static one due to three concurrent mechanisms: (i) rate sensitivity of the parent material, typically captured by Johnson–Cook or Cowper–Symonds constitutive laws; (ii) microinertial stabilization of cell walls and struts that delays elastic buckling and plastic collapse; and (iii) shock-wave-like front densification above a critical impact velocity. Experimental studies on metallic lattices and foams have consistently reported dynamic enhancement factors of the plateau stress and SEA in the range ~1.3–2.5 relative to quasi-static conditions [48,58]. Despite this well-established rate dependency, EV-oriented studies, particularly those coupling optimization algorithms with FE simulations, rarely report dynamic calibration of the cellular model and frequently extrapolate quasi-static performance indicators to crash scenarios. Future research should therefore prioritize (a) dynamic compressive characterization at intermediate strain rates (10^0^–10^2^ s^−1^) via split-Hopkinson pressure bar (SHPB) and instrumented drop-tower setups, and (b) the development of rate-dependent constitutive models for architected cellular structures, suitable for explicit crash simulations at pack and vehicle level.

From an industrial perspective, scalability and manufacturability remain major bottlenecks for advanced cellular architectures. While lattice-based, auxetic, and bio-inspired structures demonstrate excellent performance at component level, the production of complex geometries with tight tolerances, high repeatability and acceptable costs remains challenging, particularly for large-volume EV manufacturing [14,15,16,23]. Moreover, the integration of cellular structures into existing vehicle platforms introduces additional constraints related to packaging, assembly, inspection and repairability.

Based on the identified gaps, several promising research directions can be outlined:

- Development of standardized testing protocols and performance metrics for cellular-based battery protection systems;
- Increased emphasis on experimental validation, including pack-level and vehicle-level impact testing;
- Advancement of multiphysics modeling frameworks integrating mechanical, thermal and electrochemical phenomena;
- Systematic dynamic characterization of cellular architectures at intermediate strain rates and development of rate-dependent constitutive models for crashworthiness simulations;
- Exploration of hybrid solutions combining cellular architectures with conventional structural components;
- Investigation of scalable manufacturing routes, including hybrid additive–conventional processes;
- Integration of data-driven and machine learning approaches to accelerate design optimization, robustness assessment, and uncertainty quantification.

Addressing these challenges will be essential to bridge the gap between academic research and industrial implementation of cellular structures in EV battery systems. Overcoming current limitations will require coordinated efforts across modeling, experimentation, manufacturing and system integration.

Overall, while cellular structures offer significant opportunities to enhance the safety, efficiency and multifunctionality of EV battery systems, their full potential has yet to be realized. Progress in the identified research directions will play a key role in enabling next-generation EV platforms that combine advanced structural protection with industrial feasibility and regulatory compliance.

## 12. Conclusions

Cellular architectures offer a broad and complementary design space for improving EV battery-pack protection and integration, but their apparent superiority is highly sensitive to normalization, boundary conditions and system constraints. Honeycombs remain the most widely adopted industrial solution, delivering robust and cost-effective energy absorption when the loading direction is well defined, yet their anisotropy can limit performance under multiaxial and integration-driven conditions. Foams provide isotropy and attractive thermal-management potential, especially in open-cell metallic configurations, but typically exhibit moderate crash energy absorption compared with optimized periodic architectures. Lattice and TPMS-based designs enable the highest tunability and multifunctional integration potential, making them promising candidates for structural enclosures and “pack-as-a-structure” solutions; nonetheless, their adoption is still constrained by manufacturing cost, defect sensitivity and limited large-scale qualification. Auxetic and bio-inspired architectures can be particularly effective for intrusion mitigation and localized cell protection, but current evidence is dominated by idealized numerical studies and lacks consistent pack-level validation. Overall, architecture selection should be requirement-driven and system-level, balancing crash performance, deformation control, thermal pathways, manufacturability, cost and serviceability. Future progress requires standardized benchmarks, increased experimental validation at component and pack scales, and coupled mechanical–thermal–electrochemical modeling to reliably assess safety-critical failure mechanisms and enable production-ready cellular solutions for next-generation EV platforms.

## Figures and Tables

**Table 1:** Performance metrics adopted for the comparison of cellular structures in electric vehicle applications.

Metric	Symbol	Definition	Relevance for EV Applications
Relative density	$\bar{\rho }$	Ratio between cellular and solid density	Lightweight design
Specific Energy Absorption	SEA	Absorbed energy per unit mass	Crashworthiness
Peak Crushing Force	PCF	Maximum force during collapse	Structural safety
Plateau stress	$\sigma_{p}$	Average stress during collapse	Load stability
Densification strain	$\varepsilon_{d}$	Onset of densification	Energy absorption limit
Effective thermal conductivity	$k_{eff}$	Equivalent thermal conductivity	Thermal management

**Table 2:** Summary of representative numerical and experimental studies on cellular structures for EV battery pack protection. Reported performance metrics are shown as presented in the original studies and may not be directly comparable due to differences in loading conditions, normalization criteria, and modeling assumptions. Legend: NR = Not reported in source study; NA = Not applicable (optimization focuses on other parameters); PCF = Peak crushing force; ↓ = reduction observed.

Cellular Architecture	Methodology	SEA (J g^−1^)	Deformation Reduction	Stress Reduction	Key Remarks
Bio-inspired cellular structures	Review [22]	NR	NR	NR	Comprehensive review highlighting design trends and crashworthiness potential
Nature-inspired cellular structures	Numerical [32]	up to ~35	NR	NR	Large parametric screening (45 configurations) showing high SEA values
Bio-inspired honeycomb	Numerical [33]	NR	~30%	~10%	Effective deformation and stress mitigation for directional impact scenarios
Auxetic structures	ML + NSGA-II optimization [34]	baseline 3.0 → optimized 40.0	NA	NA	Optimization spans material (carbon steel → Al6061-T6), geometry (Re-entrant → Star-shaped) and thickness (1 → 2.95 mm) simultaneously
Honeycomb lattice	Numerical (explicit dynamics) [35]	NR	NR	↓ PCF	Improved load distribution and reduced peak crushing force in battery pack models

**Table 3:** Reported ranges refer to quasi-static compression tests ($\;\dot{\varepsilon }$ ≈ 10^−3^–10^−1^ s^−1^) on metallic architectures with the parent material being either aluminum alloy (Al-Si or 6xxx series), Ti-6Al-4V, 316L stainless steel or AlSi10Mg AM-grade. Values of SEA are given at 50% strain and normalized to the apparent (bounding-box) mass, unless otherwise specified in the original sources. The peak-to-plateau stress ratio (σ_peak_/σ_plateau_) is a dimensionless indicator of collapse stability based on engineering considerations: values close to 1 are typical of auxetic and TPMS sheet-based lattices, while ratios above 1.5 indicate marked elastic spikes that may compromise cell-level protection of battery packs. Relative cost is indicated as an order-of-magnitude factor with respect to baseline Al honeycomb (≈1×); absolute values depend strongly on production volume and manufacturing route. Effective thermal conductivity values refer to air-filled configurations at room temperature. Data ranges should be interpreted as indicative of the literature envelope and not as absolute limits.

Architecture	ρ*/ρs	SEA [J g^−1^]	σ_peak_/σ_plateau_	$k_{eff}$ [W/(m·K)]	Rel. Cost	Manufacturability	References
Honeycomb (Al, hexagonal, out-of-plane)	0.02–0.10	15–35	1.3–1.8	0.5–5 (along cell axis)	Low (≈1×)	High (extrusion, expansion)	[17,21,30,51]
Metallic foams (open-cell, Al)	0.05–0.20	3–15	1.1–1.3	5–15	Low (≈1×)	High (powder metallurgy, infiltration)	[18,27]
Metallic foams (closed-cell, Al)	0.10–0.30	5–25	1.2–1.5	2–8	Low–Medium	High–Medium	[18,26,51]
Lattice, strut-based (AM, stretching-dominated)	0.10–0.40	15–60	1.4–2.2	3–20	High (≈3–5×)	Low–Medium (SLM/EBM)	[15,16,48,49]
Lattice, TPMS sheet-based (AM)	0.15–0.50	20–80	1.1–1.5	5–25	High (≈3–5×)	Low–Medium (SLM/EBM)	[24,48,52,53]
Auxetic (metallic/composite)	0.05–0.25	5–40	0.8–1.2	1–5	Medium–High (≈2–4×)	Low (AM or specialized forming)	[28,29,34]

## Data Availability

No new data were created or analyzed in this study. Data sharing is not applicable to this article.
